# Expression Profiles of AQP3 and AQP4 in Lung Adenocarcinoma Samples Generated via Bronchoscopic Biopsies

**DOI:** 10.3390/jcm11195954

**Published:** 2022-10-09

**Authors:** Lukasz Jaskiewicz, Karolina Hejne, Blazej Szostak, Karolina Osowiecka, Mariusz T. Skowronski, Ewa Lepiarczyk, Anna Doboszynska, Marta Majewska, Pawel Kordowitzki, Agnieszka Skowronska

**Affiliations:** 1 Department of Human Physiology and Pathophysiology, School of Medicine, Collegium Medicum, University of Warmia and Mazury, al. Warszawska 30, 10-082 Olsztyn, Poland; 2 Department of Pathomorphology and Forensic Medicine, School of Medicine, Collegium Medicum, University of Warmia and Mazury, ul. Zolnierska 18, 10-561 Olsztyn, Poland; 3 Provincial Specialist Hospital, ul. Zolnierska 18, 10-561 Olsztyn, Poland; 4 Department of Psychology and Sociology of Health and Public Health, School of Public Health, Collegium Medicum, University of Warmia and Mazury, al. Warszawska 30, 10-082 Olsztyn, Poland; 5 Department of Basic and Preclinical Sciences, Institute for Veterinary Medicine, Nicolaus Copernicus University, ul. Gagarina 1, 87-100 Torun, Poland; 6 Department of Pulmonology, School of Public Health, Collegium Medicum, University of Warmia and Mazury, ul. Jagiellońska 78, 10-357 Olsztyn, Poland

**Keywords:** AQP3, AQP4, aquaporin, lung cancer, NSCLC, adenocarcinoma, bronchoscopy biopsies

## Abstract

Aquaporins (AQPs) are highly conserved channel proteins which are mainly responsible for the exchange of water and small molecules and have shown to play a pivotal role in the development and progression of cancer. Lung adenocarcinoma is the most common primary lung cancer seen in patients in Europe and the United States. However, in patients it is often not diagnosed until the advanced tumor stage is present. Previous studies provided strong evidence that some members of the AQP family could serve as clinical biomarkers for different diseases. Therefore, we aimed to investigate how AQP3 and AQP4 protein expression in lung adenocarcinoma (ADC) biopsy samples correlate with clinical and pathological parameters. The protein expression of AQP3 and AQP4 was analyzed based on immunohistochemical staining. AQP3 protein was observed in the cytoplasmic membrane of cancer tissue in 82% of lung samples. Significant differences in relative protein expression of AQP3 were noted between advanced age patients compared to younger counterparts (*p* = 0.017). A high expression of AQP3 was significant in cancer tissue when compared to the control group (*p* < 0.001), whereas a low AQP4 membrane expression was noted as significantly common in cancer tissue compared to non-neoplastic lung tissue (*p* < 0.001). Moreover, a low AQP4 membrane expression was positively correlated with a more advanced disease status, e.g., lymph node metastases (*p* = 0.046). Based on our findings, AQP3 and AQP4 could be used as biomarkers in ADC patients.

## 1. Introduction

Members of the aquaporin (AQP) family are expressed in different cells, tissues, and organs, and as channel proteins they are responsible for the exchange of water, small molecules, and, in consequence, they maintain cell stability and integrity [1,2,3]. Not only in physiological conditions do AQPs play a role but also in the pathophysiological conditions of the human body, when the water equilibrium is altered [4,5]. To date, four AQPs (AQP1, AQP3, AQP4, and AQP5) have been identified in the human respiratory tract [6,7,8]. Due to their before-mentioned critical imperative role in cell stability and integrity, it appears logical that AQPs, which also regulate numerous downstream effector signaling molecules, are involved in cancer development and progression. In the last decades, compelling evidence has been provided underpinning the contribution of AQPs in tumor biology [9,10,11,12,13]. In consequence, AQPs have been suggested as a possible target for cancer treatment and potential predictive biomarkers.

Lung cancer is the second most common malignant tumor worldwide, counting around 2 million cases and 1.76 million deaths per year [14]. The 5-year survival rate of lung cancer patients is only 18.4%, which reflects the lowest rate compared to other cancers. Although considerable improvement in treatment strategies for lung cancer has been reached over the last 40 years, the 5-year survival rate of lung cancer patients has minimally declined [15]. Lung epithelial tumors are commonly divided into adenocarcinoma, squamous cell carcinoma, small-cell carcinoma, large-cell carcinoma, and large-cell neuroendocrine carcinoma according to the WHO classification of lung tumors [16,17,18]. Noteworthy, lung adenocarcinoma (ADC) shows a high ability to progress rapidly and exhibits a poor prognosis [19]. Like all types of lung cancers, squamous cell carcinoma and large-cell carcinoma, are strongly associated with smoking and are diagnosed in approximately 30% and 10% of all lung cancer cases, respectively [20,21,22,23]. Previous studies partially deciphered the important role of certain AQP isoforms, for example in ADC [24], or in lung cancer cell lines [25,26,27]. More precisely, high protein levels of the AQP3 isoform were identified in proximal and terminal bronchioles and in the apical membrane of all columnar cells facing the lumen of type I and II pneumocytes [6].

Based on the before-mentioned facts, our study intended to shed light on the role of specifically AQP3 and AQP4 in ADC to extend the knowledge and clarify their value as potential predictive and/or diagnostic biomarkers. Herein, we analyzed tumor biopsy samples of 79 ADC patients at different clinical stages (CS): CS I, 3 patients; CS II, 6 patients; CS III, 16 patients; and CS IV, 54 patients. The samples were generated via bronchofiberoscopy or thoracic biopsy and further analyzed via an immuno-histochemical approach. Noteworthy, to our best knowledge, we are the first group evaluating very small biopsy specimens acquired through bronchoscopy in ADC patients with regard to the protein expression of AQP3 and AQP4.

## 2. Materials and Methods

### 2.1. Study Design and Patient Characteristics

Herein, we have retrospectively analyzed biopsy samples (material generated either from bronchoscopy or thoracic biopsies) of lung adenocarcinomas obtained at the Center for Pulmonary Diseases in Olsztyn (Poland) between 2010 and 2012. Clinical and pathologic data was generated from medical records. Biopsy tissue samples were formalin-fixed and paraffin-embedded. Two pathologists (K.H. and B.S.) examined the histological slides of pre-selected cases independently, in a blinded fashion, and re-confirmed the diagnosis of ADC according to the WHO classification. A total of 466 lung cancer cases were pre-screened, of which 165 cases were diagnosed with ADC. For the final evaluation 79 cases were taken into consideration. The control group consisted of patients without cancer, who underwent bronchoscopy or biopsy for other reasons. The ADC patients were divided according to their sex into the three age groups. All experiments of this retrospective study were performed in accordance with the Bioethics Committee localized at the School of Medicine, Collegium Medicum, University of Warmia and Mazury in Olsztyn, Poland (BC approval No. 3/2018 from 11 January 2018). Clinical and pathological features of patients included in this study are shown in Table 1.

### 2.2. Immunohistochemistry

**Figure 1:**
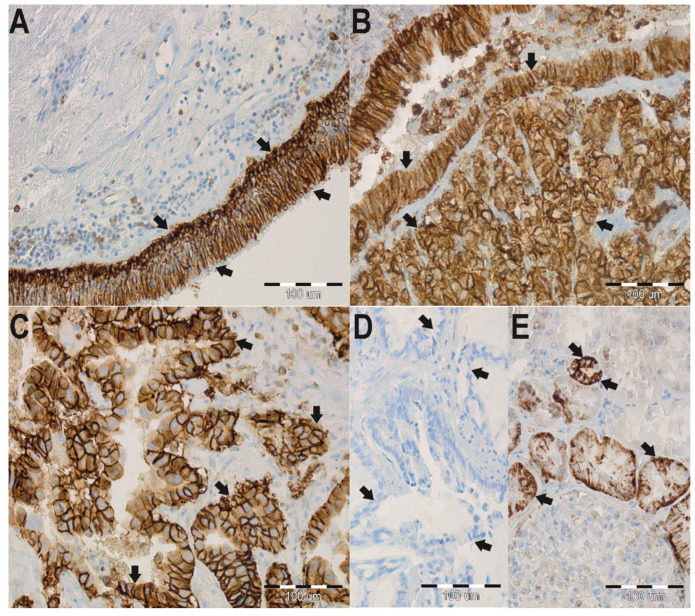
Immunohistochemistry (IHC) staining of aquaporin 3 (AQP3) in paraffin-embedded sections of the lung adenocarcinoma (ADC) from humans. Anti-AQP3 antibody labels in non-neoplastic lung tissues. Arrows indicate the localization of the basolateral plasma membranes of surface epithelium in whole plasma membranes of basal cells of the bronchus (**A**). Anti-AQP3 antibody labels in the cancer lung tissues. Arrows indicate the localization of AQP3 in the cytoplasmic membrane in the cancer cells (**B**,**C**). No staining was observed when isotype-specific immunoglobulins from non-immunized rabbits were used instead of the primary antibody (negative control) (**D**). Immunoperoxidase labeling of AQP3 from the human kidney (positive control) (**E**). The labeling is seen in the basolateral plasma membrane of the renal collecting ducts. The blue color represents a positive staining for the hematoxylin counterstain, and the brown color represents positive AQP3 stain.

**Figure 2:**
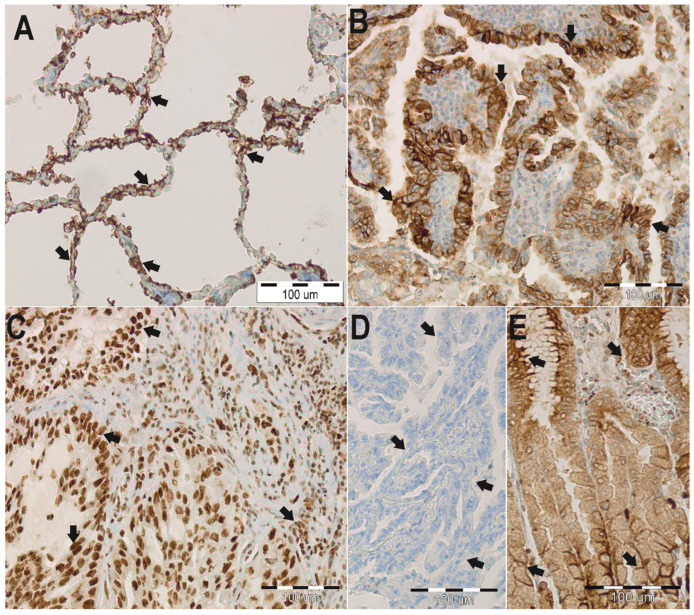
Immunohistochemistry (IHC) staining of aquaporin 4 (AQP4) in paraffin-embedded sections of the lung adenocarcinoma (ADC) from humans. Anti-AQP4 antibody labels in non-neoplastic lung tissues. Arrows indicate the localization of the type I pneumocytes and macrophages (**A**). Anti-AQP4 antibody labels in the cancer lung tissues. Arrows indicate the localization of AQP4 in the cytoplasmic membrane in the cancer cells (**B**) and aberrant nuclear reaction in cancer cells (**C**). No staining was observed when isotype-specific immunoglobulins from non-immunized rabbits were used instead of the primary antibody (negative control) (**D**). Immunoperoxidase labeling of AQP4 from the human stomach (positive control) (**E**). The labeling is seen in the basolateral plasma membrane of the surface epithelial cells and parietal cells of gastric gland cells. The blue color represents a positive staining for the hematoxylin counterstain, and the brown color represents positive AQP4 stain.

Immunohistochemical staining was performed using the BenchMark automated staining machine (Ventana Medical System, Tucson, AZ, USA). Different dilutions of antibodies, time, and temperature of incubation were tested in a pre-study. Based on this, antibodies against AQP 3 and 4 were used at a dilution of 1:200; with an affinity-purified rabbit polyclonal antibody against AQP3 (Biorbyt Ltd., orb47955, Cambridge, UK) and an affinity-purified rabbit polyclonal antibody against AQP4 (Affinity Biosciences; AF5164, Cincinnati, OH, USA). Tissue sections (thickness of 4 μm) were deparaffinized in xylene, rehydrated in three graded alcohol chambers, and treated with 3% hydrogen peroxide in methanol at room temperature for the blocking of endogenous peroxidase activity. The avidin–biotin–peroxidase technique was used for the visualization of AQP3 and AQP4 antibodies (Agilent Technologies, Inc., K5005, Dako REAL Detection System, Hamburg, Germany), followed by a chromogen detection with diaminobenzidine (DAB). As a positive control for AQP3, renal tissue samples, and for AQP4 gastric tissue samples were used (Figure 1E and Figure 2E, respectively). In addition, immunoglobulins from non-immunized rabbits were used as a negative control (dilution 1:200, Figure 1D and Figure 2D, respectively). The microscopic analysis was carried out using an Olympus light microscope (BX41, Tokyo, Japan), and pictures were taken with an Olympus XC50 (Tokyo, Japan) integrated camera.

### 2.3. Scoring System for Immunohistochemistry Results

A semiquantitative immunohistochemistry score (based on the staining intensity and the percentage of stained cells) was established according to a previously published scoring system [28,29,30,31]. Briefly, staining intensity was defined using an ordinal scale (0—no staining, 1—weak staining, 2—moderate staining, 3—strong staining), while the percentage of stained cells was measured as a continuous variable (0–100%) and was graded as follows: 0—no staining, 1—<33%, 2—34–66%, 3—>67%. The value for positively stained cells was then multiplied by the value for staining intensity. The scoring results were further evaluated by a semiquantitative approach in which AQP3 and AQP4 expression was defined as “low expression” with scores 0–2 and as “high expression” with scores 3–9. All stained slides were examined in a blinded fashion by two pathologists (K. H. and B. S.).

### 2.4. Statistical Analysis

Descriptive statistics for group characteristics were used. For the comparison between the AQPs expression profiles, and the clinicopathological findings, the χ^2^ test and *t*-student test were used. The normality of the age distribution was tested using the Shapiro–Wilk test. Survival probabilities were estimated by Kaplan–Meier method and the differences in survival median were compared using the log-rank test. Univariable and multivariable predictors of overall mortality were estimated through Cox regression analysis. Univariate variables with *p* ≤ 0.1 were included in the multivariable model. Overall survival (OS) was defined as the time from histopathological diagnosis to death. A *p*-value < 0.05 was considered to be significant. The analysis was conducted using Statistica, version 13 (http://statistica.io, accessed on 21 December 2021) TIBCO Software Inc., Krakow, Poland (2017).

## 3. Results

### 3.1. Immunohistochemistry Results for AQP3 and AQP4

**Figure 3:**
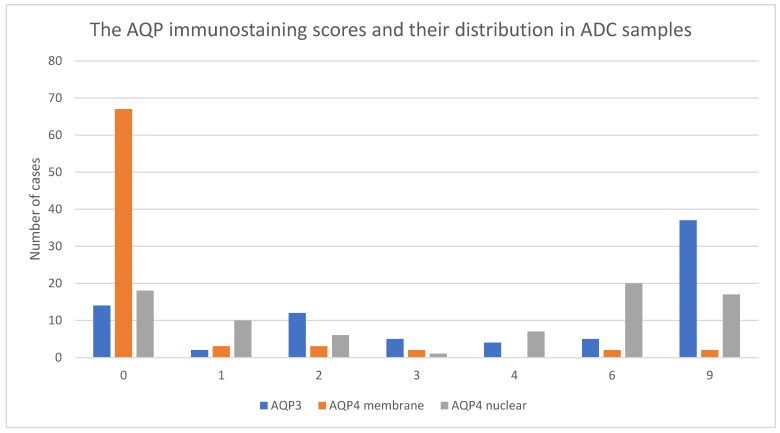
Bar diagram representing the number of lung adenocarcinoma (ADC) samples (Y axis) with low (0–2) or high (3–9) immunostaining scores (X axis) of aquaporin 3 (AQP3; blue bars), aquaporin 4 (AQP4) expressed in the cytoplasmic membrane (orange bars) or AQP4 expressed in the nucleus (grey bars).

Immunohistochemical stainings were performed to determine the localization and expression profile of AQP3 and AQP4 in ADC samples (Figure 3). Based on the analysis of non-neoplastic lung tissue, AQP3 expression was detected in the basolateral plasma membranes of surface epithelium, in plasma membranes of basal cells of the bronchus, and also in the apicolateral surfaces of type II pneumocytes and macrophages (Figure 1A). AQP4 was expressed in type I pneumocytes and macrophages. Although there was no AQP4 expression in the membranes of type I pneumocytes and macrophages, an aberrant nuclear-cytoplasmic AQP4 expression was detected (Figure 2A). In 82% of analyzed ADC samples, AQP3 was observed in the cytoplasmic membrane (Figure 1B,C).

A high immunoreactivity of AQP3 (scores 3–9) was observed in 100% of control samples, thus significantly different compared to a 65% expression in ADC samples (*p* < 0.001). When comparing the mean age of patients from the “low AQP3 expression” group with the “high AQP3 expression” group, a significant difference could be revealed (64.6 ± 8.9 years of age vs. 60.3 ± 6.5 years of age, *p* = 0.017). Interestingly, a significant (*p* = 0.017) lower relative AQP3 expression (scores 0–2) was detected in older patients (average 64.6 ± 8.9 years of age) compared to younger counterparts (average age 60.3 ± 6.5 years). It is worth noting that AQP3 expression was not influenced either by gender or tumor characteristics (Table 2). However, a relative AQP4 protein expression was observed in the cytoplasmic membrane of cancer cells in 16% of ADC samples. Furthermore, in 77% of the analyzed samples, we detected an aberrant nuclear reaction in cancer cells of varying intensity (Figure 2B,C). Interestingly, a low AQP4 expression (scores 0–2) was significantly (*p* < 0.001) more common in ADC samples (92%) than in samples of the control group (32%). Furthermore, a low AQP4 membrane expression was present in 89% of ADC samples, compared to a high AQP4 expression (scores 3–9), and it was correlated with worse cancer-related characteristics with lymph nodes metastases, which was a significant difference (*p* = 0.046). However, no differences were identified between AQP4 membrane expression and age, gender, or TNM stages (Table 2).

### 3.2. Correlation among AQPs Expression, Outcome and Clinical Factors

**Figure 4:**
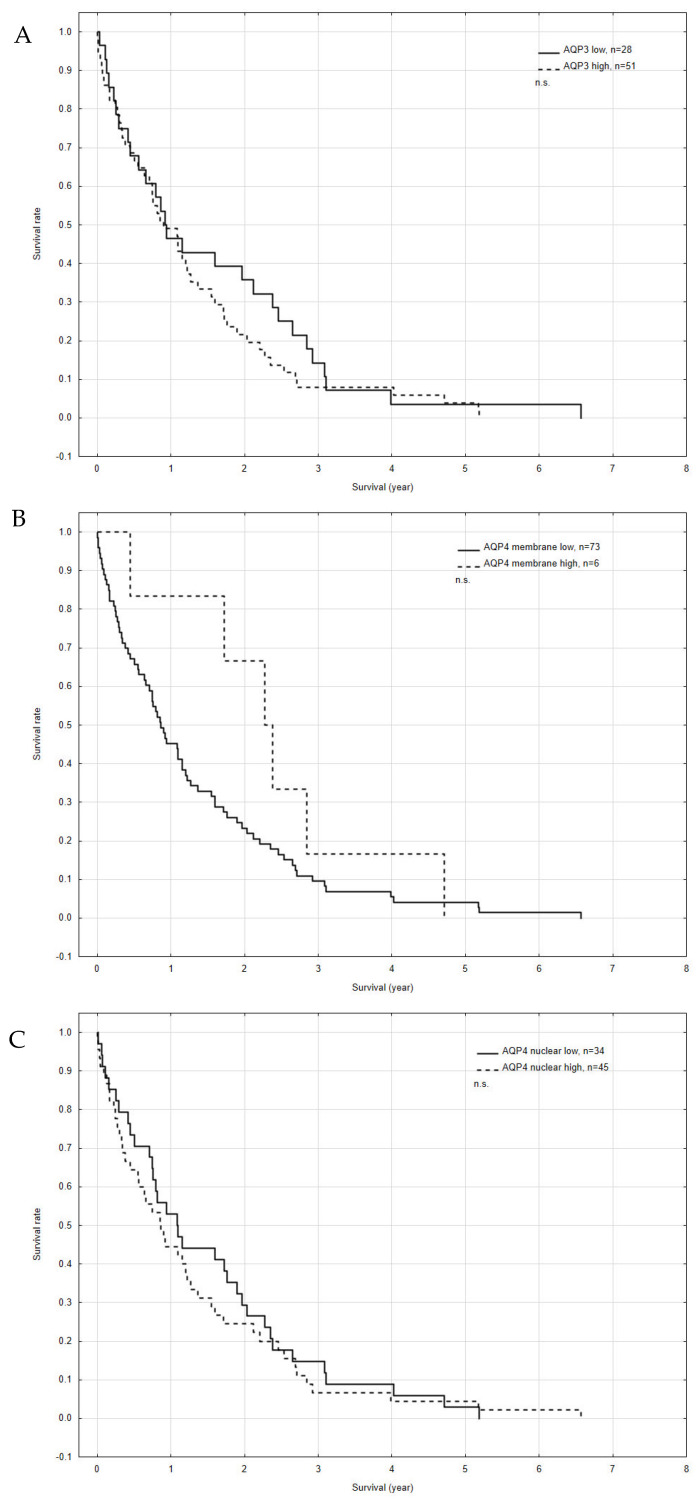
Kaplan–Meier survival curves for overall survival (OS) according to the expression of aquaporin 3 (AQP3) (**A**); aquaporin 4 (AQP4) membrane expression (**B**); and AQP4 nuclear expression (**C**). n.s.—no significance.

The 2-year overall survival (OS) rate of patients with low and high AQP3 expression did not differ significantly (36% vs. 22%, respectively, *p* = 0.32). There were no significant differences in OS among samples with low or high AQP4 membrane (23% vs. 67%, respectively, *p* = 0.09; and nuclear expression 29% vs. 25% *p* = 0.53; Figure 4A–C). In the uni- variate analysis, a significant (*p* < 0.05) influence of tumor size, distant metastases, and treatment intention on OS was detected. The more advanced the disease, the worse the prognosis for the patients (Table 3).

## 4. Discussion

Herein, we investigated the protein expression profiles of AQP3 and AQP4 in neoplastic and non-neoplastic lung tissue biopsy samples. The first difference, which is worth emphasizing, between our approach and those from previous research groups is the way of sample generation. So far, samples have been obtained during surgery from patients suffering from ADC. There is no doubt that these studies have significantly contributed to a better understanding of AQP’s role in cancers. However, biopsy samples as those in our current study have rarely been studied directly. According to standard procedures in many clinics, it is important to collect material for histological examination, once lung cancer is suspected. During this microscopic evaluation, the type and subtype of the tumor are determined, and if mandatory, further molecular tests are performed [32]. In the presented cases, bronchoscopy was a routine examination. Furthermore, biopsies taken under bronchoscopic control are often the only way to obtain specimens for the diagnostic purposes. Nevertheless, the choice of an adequate diagnostic test depends on the tumor localization [33,34].

With regard to our initial question, if AQPs could be used as reliable prognostic indicators in ADC, it has to be said that, for instance, in hepatic cancer, the immunohistochemical detection of AQP1 represents a reliable way to differentiate between cholangiocarcinoma, hepatocellular carcinoma, and metastatic colorectal carcinomas. Moreover, evaluating AQP1 concentrations in the urine of patients suffering renal cell carcinoma has shown to be a helpful biomarker [35,36]. A decreased overall survival of cutaneous melanoma patients has been linked to a positive AQP1 expression, and an increased AQP3 expression in skin cancer patients [37,38,39] and lung cancers [40] has been detected. In line with the previous mentioned studies, we detected significant (*p* < 0.001) high AQP3 expression levels in 65% of all ADC biopsies. Further, we showed that AQP3 was distinctively expressed in the cytoplasmic membrane of cancer cells in 65 of 79 lung carcinoma biopsies (Figure 1B,C), whereas in non-neoplastic lung samples, AQP3 was localized in basolateral plasma membranes of the surface epithelium (Figure 1A). Interestingly, AQP3 expression was present regardless of gender and tumor characteristics. However, no significant correlation was found between the 2-year overall survival (OS) of patients and the AQP3 expression. We observed an interesting tendency that patients with a low AQP3 expression in ADC had a better overall survival rate (31%) than patients with high AQP3 expression (50%). The before-mentioned findings are comparable to previous research in which AQP3 has been suggested to have a significant role in tumor biology as it alters cellular signaling, encourages tumor development, and contributes to proliferation, epithelial–mesenchymal transition and metastasis [13,41,42].

Concerning the question of how AQPs facilitate cell migration, it appears likely that water entry into the leading edge of the cell enhances the formation of the so-called lamellipodium [43]. So far, there is a clear consensus in the literature that AQP3 is involved in the development of lipid structures by providing glycerol and that AQP3 is involved in cell proliferation and growth by encouraging ATP and therefore may play a key role in tumor energy metabolism [44,45]. Moreover, AQP3 appears to be involved in the process of angiogenesis in lung cancer through the HIF-2α-VEGF pathway, and AQP3 does also play a role in cell invasion, partly via the AKT-MMPs pathway [40]. On contrary, Kusayama and colleagues (2011) observed that the immunostaining pattern of AQP3 in human primary squamous cell carcinoma (SCC) was dependent on cancer type since an eminent AQP3 expression was detectable in skin SCC but only a poor expression in lung SCC [46]. Furthermore, it has been shown that the suppression of AQP3 either with AQP inhibitors or AQP3-siRNA-induced cell death in the SCC cell lines [46]. The signaling pathways and the functions associated with AQPs in lung cancer have thus been studied to elucidate their contribution to tumor development.

The role of AQP4 in ADC still needs further elucidation, that is why we selected it to be the subject of our investigation [47]. Warth and colleagues (2011) identified for the first time an AQP4 expression (isoforms M1 and M23) in differentiated ADC [48]. Moreover, they emphasized the link between AQP4 and physiological lung function, which appears to decrease with advancing tumor dedifferentiation. In our study, a significantly (*p* < 0.001) higher AQP4 expression was seen in ADC samples compared to non-neoplastic samples. In lung tissue of the control group, AQP4 was localized in type I pneumocytes and macrophages, whereas in the ADC group, a different localization pattern was observed. It is still unclear which factors are responsible for the expression as well as for the distribution of AQP4. More research is needed to answer the question how the lung environment/different extracellular components affect the AQP4 expression in tumors.

Our results demonstrated that a low AQP4 membrane expression was correlated with more advanced cancer stages, meaning the presence of lymph node metastases (89% vs. 67%, *p* < 0.05, respectively). Xu and colleagues found a subsequent loss of AQP4 during carcinogenesis in gastric cancer and suggested AQP4 as a marker for normal proliferating gastric cells [49,50]. Previous reports showed that AQP4 was poorly expressed in ADC cells and overexpression of AQP4 could inhibit the migration and invasion of cancer cells through the suppressive role of miR-196b [51]. In contrast to the results of Warth and colleagues (2011), which showed that higher AQP4 expression profiles were related to a more advantageous prognosis in stage I ADC patients [48], we could not find significant differences in survival rates between the low- and high-AQP4 expression groups. Nevertheless, we are aware of certain limitations in our study. Due to the fact that our findings are based on a limited number of samples, the results should be treated with considerable caution. Moreover, the biopsy samples were generated in one hospital and the access to ADC cases was limited. Secondly, although the material has been analyzed by experienced pathologists, inter-observer variability cannot be neglected. In addition, most of the ADC patients were at an advanced disease stage, and no surgical materials were available. Importantly though, the collected material was unique research material due to its very small amount, which could be used for AQP protein staining.

## 5. Conclusions

Developing a profound and evidence-based understanding of how AQP3 and 4 are interacting with signaling pathways that are known to have a crucial relevance in lung cancer development and progression will be extraordinarily important for the detection of novel therapeutic strategies. However, the potential clinical prognostic value of AQP3 and AQP4 expression for bronchoscopy samples or material from transthoracic needle aspiration still warrants further elucidation. Nevertheless, our study showed that these specimens were adequate to determine the expression of AQPs in ADCs.

## Figures and Tables

**Table 1:** Clinicopathological features of patients diagnosed with ADC in this study.

Variables		*n*	(%)
Total number of patients		79	
Mean Age ± SD, years (range)		61.8 ± 7.7 (47–81)	
Gender			
	Female	27	(34.2)
	Male	52	(65.8)
CS			
	I	3	(3.8)
	II	6	(7.6)
	III	16	(20.2)
	IV	54	(68.4)
T			
	Tx	2	(2.5)
	T0	1	(1.3)
	T1	12	(15.2)
	T2	29	(36.7)
	T3	4	(5.1)
	T4	26	(32.9)
	no data	5	(6.3)
N			
	N0	10	(12.7)
	N1	7	(8.9)
	N2	38	(48.1)
	N3	22	(27.8)
	no data	2	(2.5)
M			
	M0	26	(32.9)
	M1	53	(67.1)
Intention to treat			
	radical	19	(24.0)
	palliative	59	(74.7)
	no consent	1	(1.3)
Radical treatment	operation + adjuvant radiochemotherapy	8	(42.1)
	radiochemotherapy	7	(36.8)
	operation	4	(21.1)
Palliative treatment	palliative chemotherapy	41	(69.5)
	palliative radiotherapy	5	(8.5)
	molecular targeted therapy	4	(6.8)
	symptomatic treatment	3	(5.1)
	no data	6	(10.1)

*n*—number; ±SD—standard deviation; CS—clinical stage; T—primary tumor; N—regional lymph nodes; M—distant metastasis.

**Table 2:** Relationship between aquaporin 3 (AQP3) and aquaporin 4 (AQP4) and clinical-/pathological features of patients diagnosed with lung adenocarcinoma (ADC) in this study.

	AQP3 Score					AQP4 Membrane Score					AQP4 Nuclear Score				
Variables	Low		High		*p*-Value	Low		High		*p*-Value	Low		High		*p*-Value
	*n*	(%)	*n*	(%)		*n*	(%)	*n*	(%)		*n*	(%)	*n*	(%)	
Mean Age ±SD (years)	64.6 ± 8.9		60.3 ± 6.5		0.017	61.6 ± 7.5		64.5 ± 9.9		0.38	62.6 ± 8.2		61.2 ± 7.3		0.44
Gender															
Female	8	(29)	19	(37)	0.44	24	(33)	3	(50)	0.40	15	(44)	12	(27)	0.11
Male	20	(71)	32	(63)		49	(67)	3	(50)		19	(56)	33	(73)	
CS															
I–II	3	(10)	6	(12)	0.39	7	(10)	2	(33)	0.21	6	(18)	3	(7)	0.31
III	8	(29)	8	(16)		15	(20)	1	(17)		6	(17)	10	(22)	
IV	17	(61)	37	(72)		51	(70)	3	(50)		22	(65)	32	(71)	
T															
T1	8	(32)	4	(9)	0.06	10	(15)	2	(50)	0.20	6	(21)	6	(14)	0.70
T2	7	(28)	22	(48)		27	(40)	2	(50)		13	(45)	16	(38)	
T3	2	(8)	2	(4)		4	(6)	0	(0)		1	(3)	3	(7)	
T4	8	(32)	18	(39)		26	(39)	0	(0)		9	(31)	17	(41)	
N															
N0	2	(7)	8	(16)	0.74	8	(11)	2	(33)	0.046	7	(21)	3	(7)	0.07
N1	3	(11)	4	(8)		5	(7)	2	(33)		5	(15)	2	(5)	
N2	14	(52)	24	(48)		37	(52)	1	(17)		14	(43)	24	(54)	
N3	8	(30)	14	(28)		21	(30)	1	(17)		7	(21)	15	(34)	
M															
M0	11	(39)	15	(29)	0.37	22	(30)	4	(67)	0.07	13	(38)	13	(29)	0.38
M1	17	(61)	36	(71)		51	(70)	2	(33)		21	(62)	32	(71)	
Intention to treat															
radical	8	(30)	11	(22)	0.43	16	(22)	3	(50)	0.13	9	(27)	10	(23)	0.70
palliative	19	(70)	40	(78)		56	(78)	3	(50)		25	(73)	34	(77)	

±SD—standard deviation; low AQP—0–2 scores, high AQP—3–9 scores.

**Table 3:** Table shows the univariate and multivariate analyses for overall survival (OS) of all patients suffering from lung adenocarcinoma (ADC).

		Univariate OS Analysis		Multivariate OS Analysis		
Variables	HR (95% CI)		*p*	HR (95% CI)		*p*
Age	1.01	(0.98–1.04)	0.54			
Gender						
Female	1.00	Reference				
Male	1.44	(0.90–2.30)	0.13			
CS						
I–II	1.00	Reference		1.00	Reference	
III	0.82	(0.35–1.93)	0.66	0.72	(0.26–1.99)	0.53
IV	2.37	(1.15–4.86)	0.02	-	-	
T						
T1	1.00	Reference		1.00	Reference	
T2	1.42	(0.70–2.87)	0.33	1.48	(0.69–3.16)	0.31
T3	3.70	(1.12–12.23)	0.03	2.79	(0.70–11.14)	0.15
T4	2.04	(0.99–4.19)	0.05	1.44	(0.62–3.33)	0.40
N						
N0	1.00	Reference				
N1	1.00	(0.38–2.64)	1.00			
N2	1.01	(0.50–2.04)	0.99			
N3	1.70	(0.80–3.63)	0.17			
M						
M0	1.00	Reference		1.00	Reference	
M1	2.54	(1.54–4.19)	<0.001	1.48	(0.42–5.23)	0.54
Intention to treat						
radical	1.00	Reference		1.00	Reference	
palliative	2.34	(1.37–4.01)	<0.001	1.27	(0.51–3.13)	0.61
AQP 3						
low	1.00	Reference				
high	1.26	(0.78–2.01)	0.34			
AQP 4 membrane						
low	1.00	Reference				
high	0.57	(0.24–1.31)	0.18			
AQP 4 nuclear						
low	1.00	Reference				
high	1.15	(0.73–1.80)	0.54			

HR—hazard ratio; CI—confidence interval; low aquaporin (AQP)—0–2 scores; high AQP—3–9 scores.

## Data Availability

Data are available upon request.
